# Supplementary material for: Monoclonal Antibodies against SARS-CoV-2 Infection: Results from a Real-Life Study before the Omicron Surge
Source: Vaccines (Basel). 2022 Nov 10;10(11):1895. doi: 10.3390/vaccines10111895 (PMC9693092; doi:10.3390/vaccines10111895)
Supplement: Supplementary file 1 [file vaccines-10-01895-s001.zip › vaccines-1951726-supplementary.pdf]

**Supplementary Table S1.** Comparisons of clinical and laboratory parameters between T0 and T1

| | **T0** | **T1** |
|---|---|---|
| **Oxygen supplementation needed** (n, %) | 34 (18.4) | 14 (7.6) |
| **P/F ratio** (median, IQR) | 462 (452-467) | 467 (457-471) |
| **WBC** (cell/μL; median, IQR) | 6360 (4450-8420) | 7480 (6165-10150) |
| **Lymphocyte count** (cell/μL, median, IQR) | 1160 (845-1815) | 1640 (1170-2260) |
| **Fibrinogen** (mg/dl; median, IQR) | 387 (313-468) | 355 (275-437) |
| **D-dimer** (ng/ml; median, IQR) | 717 (419-1374) | 867 (459-1450) |
| **CRP** (mg/l) | 21.8 (5.9-54.8) | 5.5 (2.6-18.4) |
| **LDH** (U/l) | 218 (186-284) | 209 (184-267) |

P/F: perfusion/fraction of inspired oxygen. WBC: white blood count; CRP: c-reactive protein; LDH: lactate dehydrogenase
